# A major FT/TFL1 regulatory locus (*Meflwr13*) controls flowering time in cassava and provides validated markers for accelerated breeding

**DOI:** 10.3389/fpls.2026.1741780

**Published:** 2026-02-17

**Authors:** Adriana Bohorquez-Chaux, Camilo E. Sánchez-Sarria, Carmen A. Bolaños-Chaguendo, Nelson Morante, Sandra Milena Salazar, Winnie Gimode

**Affiliations:** Cassava Program, International Center for Tropical Agriculture (CIAT), Palmira, Colombia

**Keywords:** cassava, flowering, FT, marker-assisted selection, *Meflwr13*, quantitative trait locus, TFL1

## Abstract

**Introduction:**

Flowering in cassava (*Manihot esculenta* Crantz) is crucial for botanical seed production in breeding programs, but genetic improvement is severely hindered by highly variable, late, or absent flowering in many farmer-preferred genotypes. This challenge prolongs breeding cycles and necessitates costly, labor-intensive flower induction technologies. To overcome these challenges, we aimed to dissect the genetic architecture of this trait and develop molecular markers to facilitate marker-assisted selection (MAS).

**Methods:**

Quantitative trait locus (QTL) mapping was conducted in an F₂ population (AM1588) using a categorical 0–2 scoring scale across five time points (4, 6, 7, 8, and 9 months after planting [MAP]).

**Results:**

We identified QTL on chromosomes 1, 7, 13, and 16 with a stable and highly significant QTL on chromosome 13 (Meflwr13), reaching a maximum LOD of 20.82 and explaining up to 42.63% of the phenotypic variation. Fine mapping of Meflwr13 revealed a complex regulatory hub containing the antagonistic master floral switch genes, FLOWERING LOCUS T (FT) and TERMINAL FLOWER 1 (TFL1), along with key transcriptional modulators, including WRKY75, AP2/ERF and TEOSINTE BRANCHED 1 transcription factors.

**Discussion:**

This molecular architecture strongly suggests that flowering time in this population is controlled by the balance of promoting and repressing factors at this locus. To facilitate direct application, we validated key single nucleotide polymorphisms (SNPs) from Meflwr13 in an independent panel of 304 breeding progenitors. Three SNPs (C13_889929, C13_634483, and C13_658450) exhibited a dominant segregation pattern and showed favorable performance metrics, confirming their predictive power across diverse genetic backgrounds. These validated dominant markers provide breeders with an efficient, cost-effective tool for MAS, enabling rapid screening of seedlings in the nursery. Utilization of these markers will significantly accelerate the production of new, superior cassava varieties.

## 1 Introduction

Cassava (*Manihot esculenta*, Crantz) is a vital root crop globally, serving as a primary food source, animal feed, and as raw material for various industries (Parmar et al., 2017). Its resilience, including adaptability to diverse agroecologies, tolerance to marginal climate and soil conditions, and a flexible harvest window, makes it an essential food security crop, driving increasing global production, particularly in tropical countries (Nanbol and Namo, 2019; Jarvis et al., 2012). Farmers often favor erect, non-branching clones, as these varieties facilitate management and mechanization, yield more planting material, and are easier to transport and store. The longer stems also store more water, potentially enhancing the crop’s resilience to irregular rainfall associated with climate change (Ceballos et al., 2012).

While cassava is primarily propagated vegetatively from stem cuttings, rapid genetic improvement relies on sexual reproduction to introduce genetic variation. The breeding progress in cassava has been limited by factors including poor and asynchronous flowering, low seed set per cross, long cropping cycles, and low multiplication rates (Ceballos et al., 2012). These factors collectively hinder rapid genetic gain, underscoring the critical need to expand knowledge of the genetics and inheritance of flowering traits to overcome some of these challenges (Baguma et al., 2024).

Cassava is a monoecious species that produces both male and female flowers on the same plant. Male flowers are much more abundant and appear in the upper section of the inflorescence. Female flowers, on the other hand, grow on the lower (proximal) branches of the inflorescence, and anthesis occurs about 14 days earlier than that of the male flowers, a condition known as protogyny (Perera et al., 2013; Ramos Abril et al., 2019). Pollination is mainly facilitated by outcrossing via bees, although some self-pollination can occur, but the subsequent fertilization and seed set is generally inefficient (Ramos Abril et al., 2019). The inflorescence consistently emerges at the tip of the growing stem. Bud sprouting beneath the inflorescence enables the plant to continue growing, allowing it to flower first and then develop new branches, with every flowering event structurally leading to the formation of new branches. This establishes a close association between flowering and plant branching patterns: early-flowering genotypes tend to be shorter and more highly branched, while late-flowering genotypes are typically erect with minimal branching. The time and frequency of flowering is highly variable and is influenced by both genetic and environmental factors, with some genotypes flowering multiple times beginning as early as two to three months after planting (MAP), and others flowering rarely, very late or not at all (Pineda et al., 2020).

The timing of flowering is a critical agricultural trait and quantitative trait loci (QTL) associated with it have been identified across several crops (Ducrocq et al., 2009; Maheswaran et al., 2000; Thomson et al., 2003; Yan et al., 2006; Yano et al., 2001; Lu et al., 2014; Mao et al., 2017; Gimode et al., 2020; Molla, 2022; Wu et al., 2023). In many species, floral transition is regulated by major pathways including photoperiodic, autonomous, vernalization, hormonal and age-dependent pathways, with the FLOWERING LOCUS T (FT) gene being a key regulator (Amasino, 2010; Srikanth and Schmid, 2011; Song et al., 2024). Cassava possesses conserved genes for flowering, including FT, GIGANTEA (GI), CONSTANS (CO), TERMINAL FLOWER 1 (TFL1), and FLOWERING LOCUS D (FD) (Adeyemo et al., 2017; Wu et al., 2024). A global transcriptome analysis in cassava revealed developmental transitions in leaves and buds. Mature leaves showed two transcriptional stages: younger leaves (2–3 MAP) had low FT and GI expression, while older leaves (after 4 MAP) had higher levels that promoted flowering. Buds displayed three stages: early buds expressed TFL1, APETALA 1 (AP1), and SUPPRESSOR OF OVEREXPRESSION OF CONSTANS 1 (SOC1) before FT appeared in leaves. FT and GI increased with leaf age, indicating age-dependent regulation, while FD stayed constant, showing buds were ready to respond. AP1 activation in buds coincided with FT induction, confirming FT–FD interactions triggering flowering (Behnam et al., 2021). Significantly, overexpression of Arabidopsis FT in cassava successfully triggers earlier flowering and increased branching, underscoring the FT gene’s central role in regulating this process (Adeyemo et al., 2011, 2017, 2019).

Cassava flowering is highly sensitive to environmental signals, particularly photoperiod (day length) and temperature. Extending the photoperiod reduces the time until flowering begins and boosts flower production. The expression of cassava FT homologs (MeFT1 and MeFT2) is photoperiod-dependent, with MeFT2 expression directly influenced by day length (Adeyemo et al., 2019). Physiological regulation involving growth regulators and photoperiod has also been suggested by successful grafting experiments that induce early and abundant flowering (Silva Souza et al., 2018).

Previous genomic efforts have successfully identified quantitative trait loci (QTL) associated with flowering proxies, such as height to first branch (Zhang et al., 2018a) and branching levels (Baguma et al., 2024). However, the specific genetic architecture controlling the timing and presence/absence of flowering remains poorly defined. To directly overcome this limitation, this study aimed to conduct a QTL mapping analysis for flowering time in an F_2_ population. The objective was to dissect the genetic basis of this critical trait, identify major QTL and candidate genes that govern flowering, and develop molecular markers to enable marker-assisted selection (MAS). Successful identification of these markers will provide breeders with an efficient tool to rapidly screen seedlings, ultimately accelerating the production of new, superior cassava varieties.

## 2 Materials and methods

### 2.1 Plant material

The F_2_ population (AM1588) used in this study originated from the self-pollination of an F_1_ plant (CM8996-199) previously developed for genetic mapping of resistance to whiteflies (Bohorquez-Chaux et al., 2025). The two parents from which the F_1_ originated (ECU72 and COL2246) exhibited contrasting flowering phenotypes, with ECU72 male sterile, barely producing any male flowers. The CM8996–199 F_1_ however, produced both male and female flowers. The mapping population consisting of 109 individuals was first evaluated in 2020, then in 2025. This population was established at the International Center for Tropical Agriculture (CIAT) in Palmira, Colombia in a single diagonal arrangement with five plants per genotype. While the specific field plots differed between years to adhere to crop rotation practices, they were located within the same station featuring similar soil characteristics and environmental conditions. Similar agronomic management regimes were applied in both years. For validation, progenitors comprising 304 diverse landraces and breeding clones from CIAT breeding program were utilized.

### 2.2 Phenotyping

Flowering data were collected for all clones of the mapping population at 4 months after planting (MAP) in 2020 and four consecutive time points in 2025: 6, 7, 8, and 9 MAP. Phenotyping in 2025 was performed blind to genotype identity since scoring was decoded post-evaluation. At each time point, we recorded the independent presence of male and/or female flowers separately. This detailed descriptive data was then synthesized into a categorical trait score for each plant, on a 0–2 scale as follows: 0 = absence of flowering; 1 = onset of flowering (without clarity on whether the resulting flower will be either male or female); and 2 = presence of flowering (**Supplementary Figure S1**). To obtain the score of each accession, Best Linear Unbiased Predictions (BLUPs) adjusted by the grand mean were calculated from the five individual plant scores. The accessions and their corresponding flowering scores are summarized in **Supplementary Table S1**. Spearman’s correlation coefficient was applied to estimate correlations among the different time points (MAP). Phenotyping of the validation population was done at 6 MAP.

### 2.3 QTL mapping and candidate gene identification

Using the previously constructed linkage map on this AM1588 F_2_ mapping population (Bohorquez-Chaux et al., 2025), QTL mapping was performed using BLUPs for five datasets, corresponding to flowering evaluated at 4 (2020), 6, 7, 8, and 9 MAP (2025). Composite interval mapping (CIM) (Zeng, 1994) was performed in WinQTLCart 2.5 (Wang et al., 2007), with significance thresholds determined by 1,000 permutation tests (α = 0.05) (Churchill and Doerge, 1994). Analyses were run with a 10 cM window, model 6, a 1 cM walk speed, and five marker cofactors selected via forward–backward regression. Candidate genes within 2-LOD intervals of significant QTLs were identified using the *Manihot esculenta* v6 genome (https://phytozome-next.jgi.doe.gov/info/Mesculenta_v6_1) which the genetic map was based on. From the list of genes in these intervals, candidate genes were prioritized based on functional annotations and literature searches for homologs of known flowering-time regulators, previously characterized in model species.

### 2.4 Marker validation

Markers closest to the peak regions were selected for validation. Since the region on chromosome 13 was highly significant, we identified three other markers around the peak region. The significant markers were validated in the cassava progenitors (*N* = 304) exhibiting variable flowering. The allele frequencies and quality of these single nucleotide polymorphisms (SNPs) were assessed using a metric that estimates false positive (FPR) and false negative (FNR) rates (Bohorquez-Chaux et al., 2025; Mbanjo et al., 2024; Platten et al., 2019). Favorable alleles in the homozygous state were identified in genotypes with a score of 2 (presence of flowering). In contrast, unfavorable alleles in the homozygous state were identified in genotypes with a score of 0 (absence of flowering). The validation population and corresponding flowering scores and marker data are summarized in **Supplementary Table S2**.

## 3 Results

### 3.1 Phenotypic variation of the mapping and validation populations

For the 4 MAP evaluation in 2020 and the 6, 7, 8 and 9 MAP evaluation in 2025, the presence of male and female flowers was perfectly correlated (in all cases, r^2^ > 0.99, *p* < 0.001); whenever flowering occurred, both male and female flowers were present. Therefore, we analyzed the trait as general flowering. The calculated correlations (r^2^) among the different months were between 0.54 - 0.95 with the least correlation between 4 MAP and 9 MAP, and the highest observed correlation between 8 MAP and 9 MAP (**Supplementary Figure S2**). At 9 MAP, when evaluations were completed in the AM1588 F_2_ population, 62 genotypes (67.6%) exhibited both female and male flowers (score 2), seven genotypes (7.63%) showed the onset of flowering (score 1), and 40 genotypes (43.6%) displayed no flowering (score 0). The validation population, comprising 304 parental genotypes, was evaluated at 6 MAP using the same 0–2 scale applied to the AM1588 F_2_ population. Among the 304 parental genotypes evaluated, 148 (48.7%) exhibited both female and male flowers (score 2), 39 (12.8%) showed the onset of flowering (score 1), and 117 (38.5%) exhibited no evidence of flowering (score 0) (**Supplementary Table S2**).

### 3.2 QTL identification

**Figure 1:**
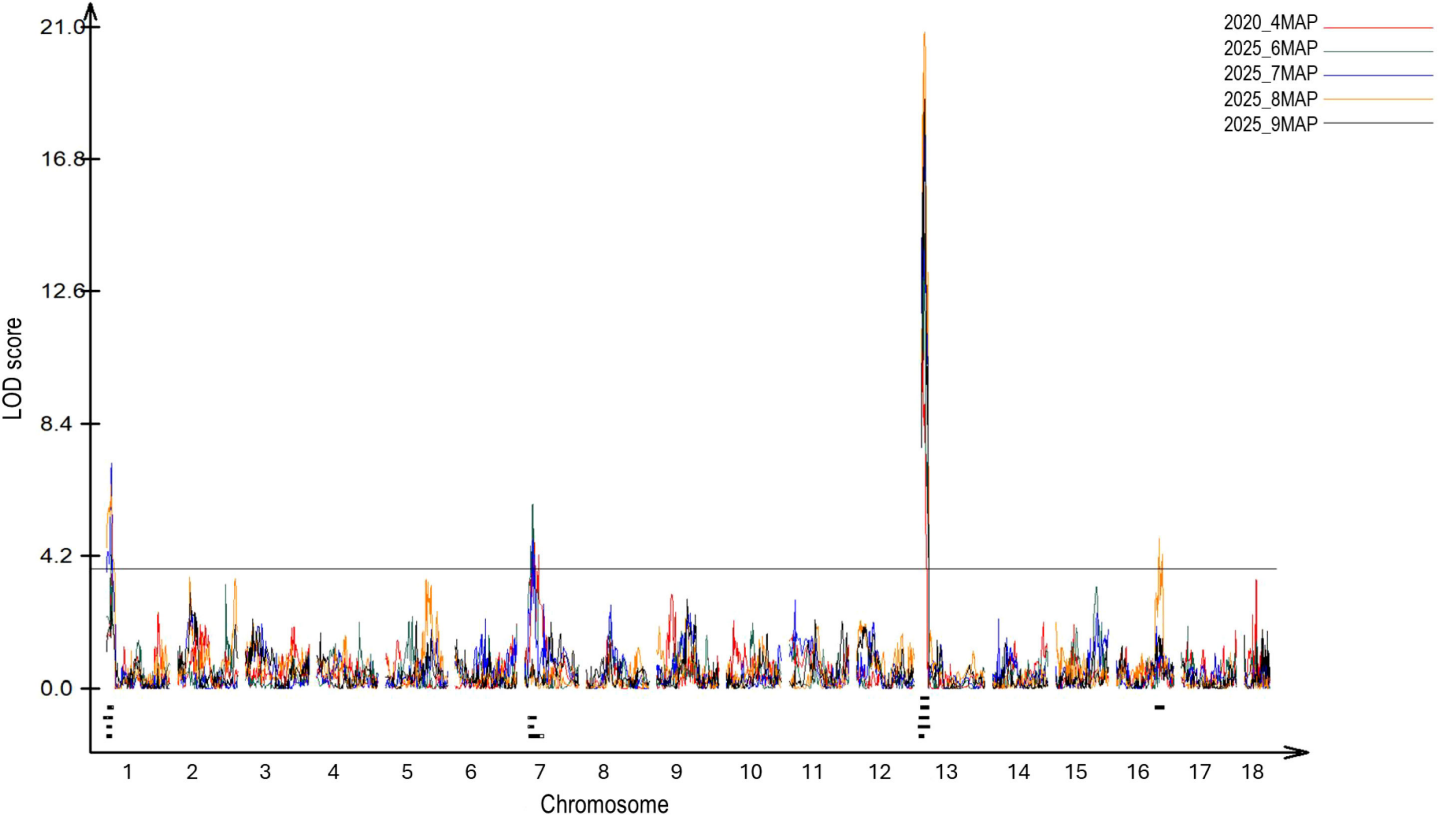
QTL associated with flowering in the AM1588 F_2_ cassava population (*N* = 109) for 4, 6, 7, 8 and 9 months after planting (MAP). The solid black horizontal line represents the significance threshold (α = 0.05) determined by 1,000 permutations. The 2-LOD support intervals for significant QTL detected on chromosomes 1, 7, 13 and 16 are indicated by short black lines below the peaks, along the x-axis.

For the five traits mapped in the AM1588 F_2_ population, QTL were detected on chromosomes 1, 7, 13 and 16, with phenotypic variance explained (R²) values ranging from 5.45% to 42.63% (**Figure 1**, **Table 1**). Among these, the QTL on chromosome 13 (*Meflwr13*) was consistent and overlapped across all five traits, while *Meflwr1* and *Meflwr7* were present in four and three traits, respectively. *Meflwr16* was only identified at 8MAP. *Meflwr13* exhibited the highest LOD scores (maximum LOD = 20.82), and phenotypic variance explained (R^2^ = 42.63%). **Table 1** summarizes the detected QTL for each trait along with their corresponding LOD scores and R^2^ values.

**Table 1:** Quantitative trait loci (QTL) associated with flowering in the AM1588 F_2_ cassava population at different months after planting (MAP) and the corresponding 2-LOD support interval.

Trait	QTL name	Chromosome	Peak (cM)	LOD^a^	Additive^b^	Dominant^c^	2-LOD interval (cM)^d^	Left flanking marker (Mb)	Right flanking marker (Mb)	R^2^ (%)^e^
2020_4MAP	*Meflwr1*	1	11.31	4.08	0.2791	0.0333	9.3-12.3	C01_6648455	C01_7051087	8.52
2020_4MAP	*Meflwr7*	7	19.63	4.61	-0.1783	0.3325	19-20.3	C07_2139477	C07_2189673	13.18
2020_4MAP	*Meflwr13*	13	2.81	11.38	0.5179	0.1501	1.4-4.4	C13_643226	C13_803536	20.51
2025_6MAP	*Meflwr1*	1	11.31	4.25	0.3195	-0.0873	9.1-12.3	C01_6648455	C01_7051087	10.17
2025_6MAP	*Meflwr7*	7	20.21	6.69	-0.396	-0.0756	18-20.8	C07_2095785	C07_2189673	9.32
2025_6MAP	*Meflwr13*	13	1.41	14.6	0.6933	-0.1178	0.2-17.4	C13_643226	C13_2443369	40.91
2025_7MAP	*Meflwr1*	1	12.91	3.91	0.5252	-0.0048	0-13.9	C01_4531869	C01_11246886	13.26
2025_7MAP	*Meflwr7*	7	20.3	4.72	-0.4144	-0.1157	17.4-21.8	C07_2095785	C07_2206413	5.45
2025_7MAP	*Meflwr13*	13	7.01	17.67	0.8318	-0.0089	6-17.2	C13_803692	C13_2495605	42.63
2025_8MAP	*Meflwr1*	1	12.31	6.48	0.4406	0.0489	11.3-16.3	C01_7120920	C01_11536977	10.25
2025_8MAP	*Meflwr13*	13	7.01	20.82	0.8843	0.4502	5.7-10.1	C13_643226	C13_2443369	21.8
2025_8MAP	*Meflwr16*	16	109.41	4.77	0.1842	-0.4182	107.4-111.7	C16_24938443	C16_25368366	9.44
2025_9MAP	*Meflwr13*	13	7.31	18.7	0.9372	0.4702	5.8-9.5	C13_803692	C13_2016918	22.88

a Logarithm of odds ratios at the position of the peak.

b Additive effect of QTL.

c Dominance effect of QTL.

d The QTL interval on genetic map.

e Percent of phenotypic variance explained by the QTL.

### 3.3 Marker association in the F_2_

**Figure 2:**
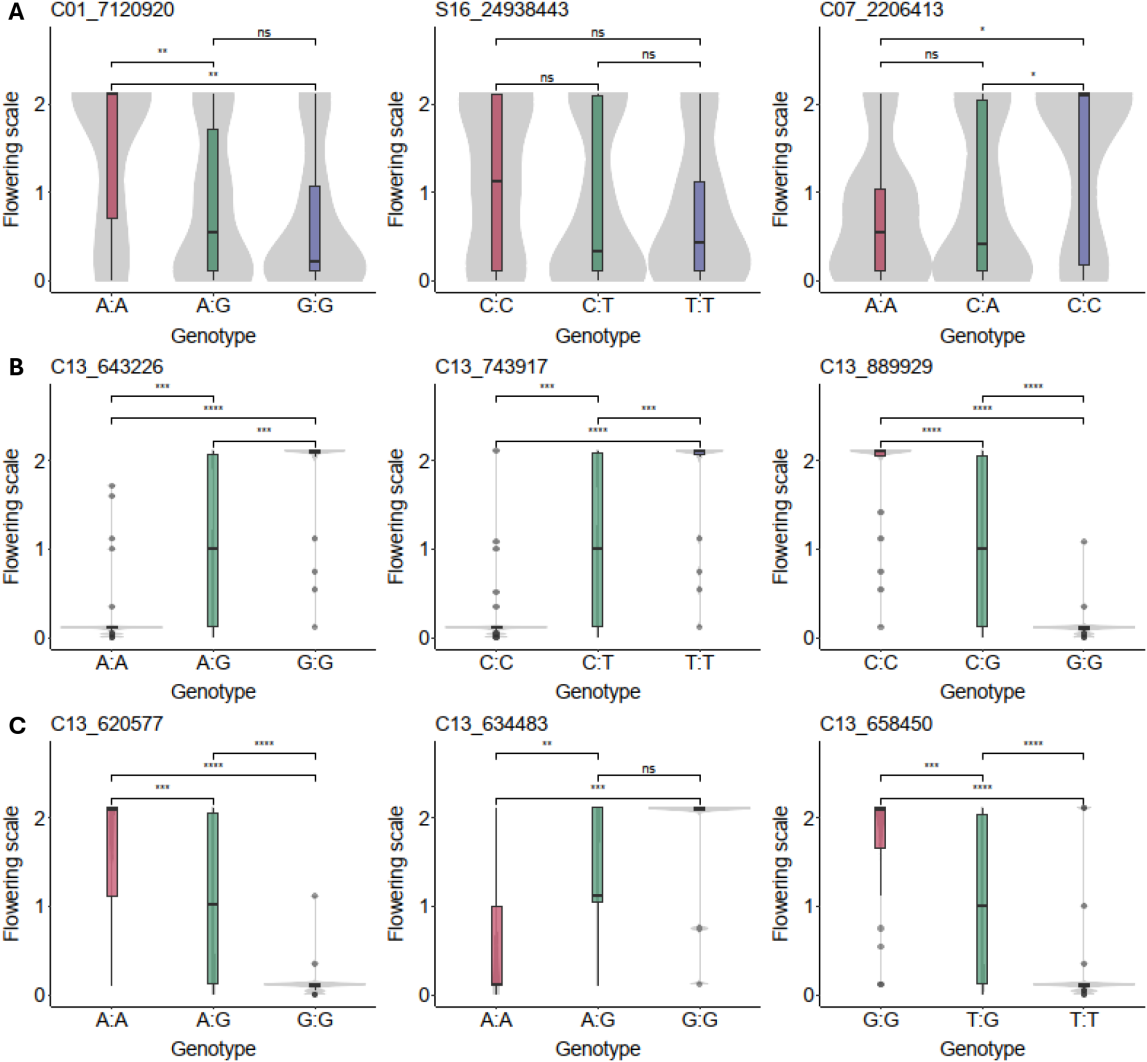
Marker-trait association on the AM1588 F_2_ cassava mapping population (*N* = 109). **(A)** Peak SNPs identified on chromosomes 1, 7, and 16. **(B)** Peak SNPs on the major QTL *Meflwr13*. **(C)** Additional SNPs fine-mapping the *Meflwr13* interval. Plots are presented as boxplots overlaid on violin plots to show data distribution, with the different colors (red, green and purple) representing the three genotype classes per SNP. The bold line within the box represents the median, while the box limits indicate the upper and lower quartiles, and whiskers extend to 1.5 times the interquartile range. *, **, ***, **** significant at *p* ≤ 0.05, *p* ≤ 0.01, *p* ≤ 0.001, *p* ≤ 0.0001, respectively; ns, non-significant (*p* > 0.05).

To validate the QTL identified in the analysis, selection focused on SNPs located at or nearest to the peak regions. **Figure 2** illustrates the marker-trait association across the candidate genomic regions. For the minor QTL regions, one representative SNP was selected from the peak area of chromosomes 1, 7, and 16. As shown in **Figure 2A**, these markers exhibited lower association with the trait. Three SNPs were selected closest to the peak of the major QTL on chromosome 13 (*Meflwr13*) (**Figure 2B**), along with a further three markers selected based on their proximity to the peak markers (**Figure 2C**), allowing for a finer view of the locus. The analysis revealed that the SNPs on *Meflwr13* exhibited the strongest association with the trait, displaying low *p* values consistent with a major effect locus, in contrast to the SNPs on the other QTL. These results confirm the high potential of this locus for marker-assisted selection (MAS).

### 3.4 Marker validation on the progenitors

**Figure 3:**
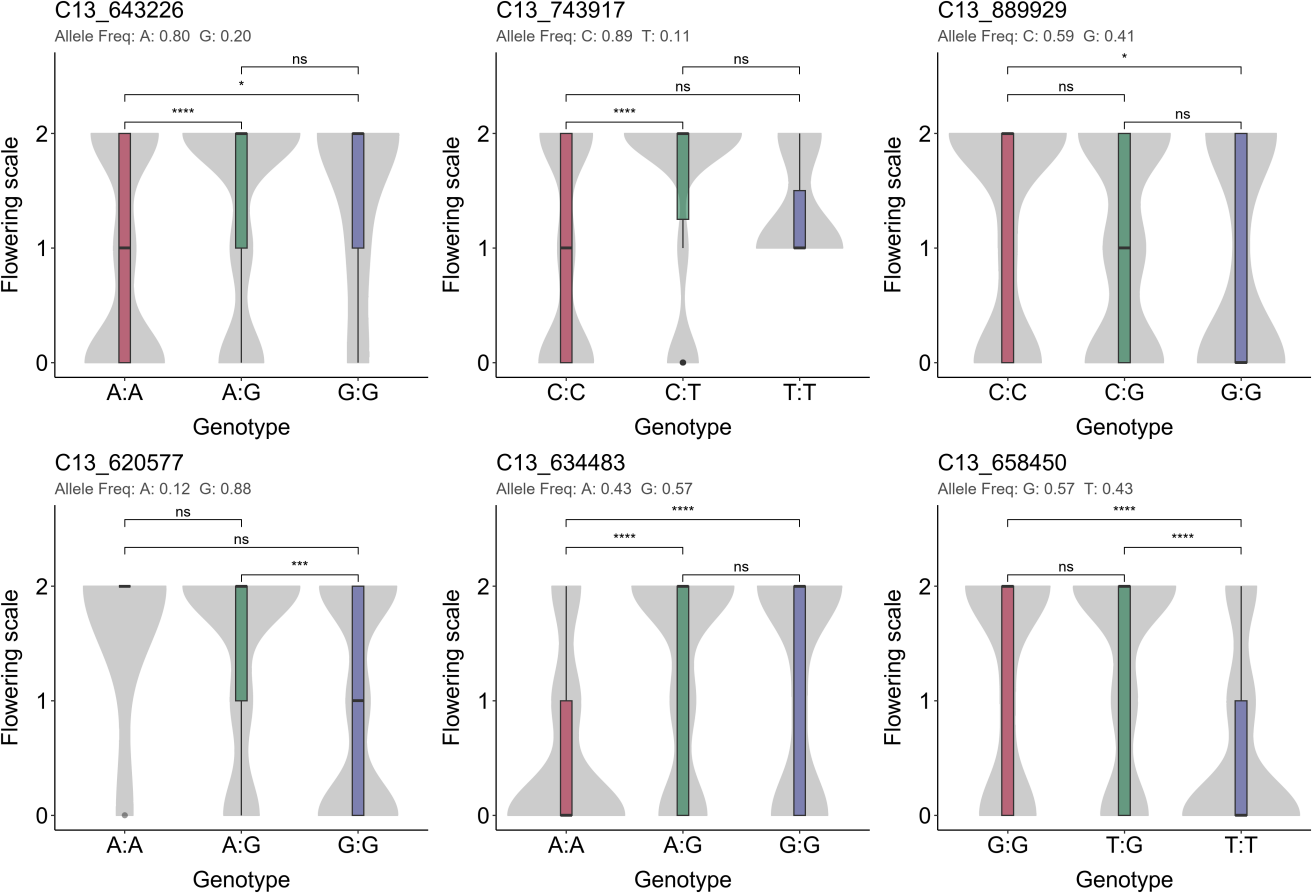
Validation of six SNPs located in *Meflwr13* on the progenitor population (*N* = 304). Phenotypic distribution of the marker genotype classes is represented by colored boxes (red, green, and purple). The outer gray violin represents the density estimation of the phenotypic distribution. Inside, boxplots display the median (bold horizontal line) and the interquartile range (box edges); whiskers extend to 1.5 times the interquartile range. *, ***, **** significant at *p* ≤ 0.05, *p* ≤ 0.001, *p* ≤ 0.0001, respectively; ns = non-significant (*p* > 0.05).

The six *Meflwr13* SNPs were further evaluated for their potential use in MAS by checking their allele frequencies and phenotypic association in the breeding progenitors of diverse backgrounds (**Figure 3**). The six SNPs exhibited dominant allelic effects as no significant difference was observed between genotypes homozygous for the favorable allele and heterozygotes. In contrast, the homozygous unfavorable genotype (phenotypic score of 0) was significantly distinct in all cases, indicating that the unfavorable allele could be effectively selected against during MAS. Notably, markers C13_634483 and C13_658450 showed the strongest differentiation between homozygous genotypes carrying the favorable and those with the unfavorable alleles (*p* ≤ 0.0001) in the progenitors. A total of 78.2%, 64.4%, and 76.8% of the evaluated genotypes carried at least one copy of the favorable alleles (C, G, and G) for markers C13_889929, C13_634483, and C13_658450, respectively. In contrast, for C13_643226, C13_743917, and C13_620577, only 45.0%, 33.4%, and 34.5% of the genotypes carried at least one copy of the favorable alleles (G, T, and A, respectively). C13_889929, C13_634483, and C13_658450 showed acceptable FPR and FNR values, while the other three had very high FNR values (**Table 2**). Based on validation in both the AM1588 F_2_ mapping population and the progenitor validation population, these three SNPs were identified as the most promising markers for selection.

**Table 2:** Frequencies of the six selected markers of the major *Meflwr13* QTL.

Marker in v6.1	Marker in v8.1	Chromosome	Favorable allele	Heterozygote	Unfavorable allele	% Homozygous major allele	% Heterozygous	% Homozygous minor allele	FPR (%)	FNR (%)
C13_643226	C13_1027778	13	G	GA	A	8.0	37.0	54.0	9.4	63.5
C13_743917	C13_1129245	13	T	TC	C	5.6	27.8	65.4	6.3	78.6
C13_889929	C13_1275797	13	C	CG	G	33.9	44.3	20.8	35	19.7
C13_620577	C13_1005133	13	A	AG	G	7.5	27.0	60.4	8	76.7
C13_634483	C13_1019035	13	G	GA	A	27.6	36.8	31.5	28.4	20
C13_658450	C13_1042992	13	G	GT	T	30.8	46.0	22.5	32.2	20.7

For each marker, the false positive rate (FPR) and false negative rate (FNR) are included, along with the SNP positions on v8.1 of the cassava reference genome.

### 3.5 Candidate gene identification

**Figure 4:**
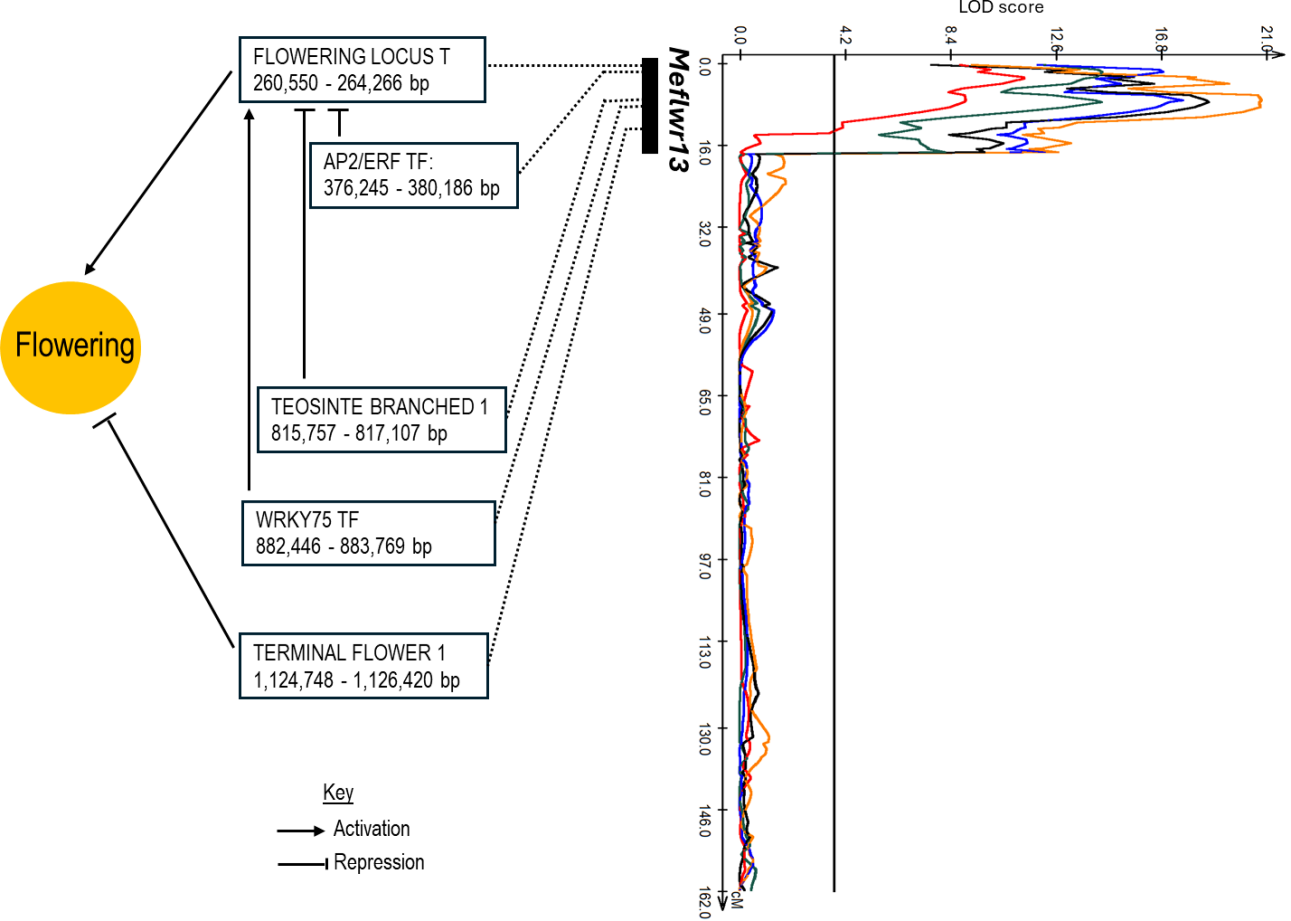
Candidate gene architecture of the major *Meflwr13* locus for flowering time in cassava. The red, green, blue, orange, and black lines represent the QTL trend lines for 4 (2020), 6, 7, 8, and 9 MAP, respectively. Boxes list the primary flowering related genes located within the QTL region and their genomic positions in base pairs (bp).

Further examination of *Meflwr13* was conducted to pinpoint potential candidate genes (**Supplementary Table 3**). Because the locus mapped to the proximal end (start) of the chromosome, the research focused on the region spanning from 0 Mb to the right-most flanking marker, located at approximately 2.5 Mb (2,495,605 bp). Flowering-related genes were identified within this region (**Figure 4**), including two major genes of interest: Manes.13G000800 (260 kb) which encodes a FLOWERING LOCUS T (FT) gene and Manes.13G011900 (1.12 Mb) which encodes a TERMINAL FLOWER 1 (TFL1) gene. The SNP explaining the highest phenotypic variation in the mapping population was C13_889929_G. This SNP mapped on an intron of a protein of unknown function (DUF1664) and is located only 6 kb from Manes.13G009100, a WRKY transcription factor (TF). WRKY TFs are implicated in modulating flowering pathways to regulate flowering time (Song et al., 2024). Interestingly, an Apetala-like ethylene-responsive transcription factor {AP2/ERF TF: Manes.13G001800 (376 kb)} and TEOSINTE BRANCHED 1 {TB1: Manes.13G008300 (815 Mb)} were also identified in the region. AP2/ERF TFs are known to repress flowering by direct repression of FT expression (Sgamma et al., 2014; Yant et al., 2010), in addition to their involvement in determining the identity of flower organs (Luo et al., 2024; Wang et al., 2023). TB1 also interacts with FT to delay flowering (Colleoni et al., 2024; Feng et al., 2022; Mimida et al., 2011). On *Meflwr1*, an APRR5 response regulator of two component system (Manes.01G043200) that controls flowering time was also identified in the peak region (7.05 Mb). Genes within the various identified QTL that have been previously reported as being involved in flowering time, male sterility/fertility, or gametophyte and pollen development in other species are summarized in **Table 3**.

**Table 3:** Putative candidate genes linked to flowering within the identified genomic loci.

Trait (MAP)	Chr	Gene ID	Gene start to end position	Protein name	References	Species and biological function
4, 6, 7, 8, 9	1	Manes.01G043200	7046185-7051604	two component response regulator-like APRR5	Sato et al., 2002; Hayama et al., 2017	In *Arabidopsis*, APRR5 regulates FLOWERING LOCUS T (FT) expression through its interaction with key components of the circadian clock and flowering pathway. APRR5 stabilizes the CONSTANS (CO) protein, that activates FT expression under long-day photoperiods.
4, 6, 7	7	Manes.07G022400	2093407-2096799	TCP domain containing protein	Colleoni et al., 2024; Dixon et al., 2018; Niwa et al., 2013	Key regulator of inflorescence architecture and flowering transitions. In *Arabidopsis*, plays a role in delaying floral transition by interfering with FT function in the apical meristem. In wheat, interacts with FT and coordinates the formation of axillary spikelets during the vegetative-to-floral transition, affecting the timing and structure of flowering.
4, 6, 7, 8, 9	13	Manes.13G000800	260550-264266	Protein FLOWERING LOCUS T	Ahn et al., 2006; Colleoni et al., 2024; Fu et al., 2025; Kardailsky et al., 1999; Kobayashi et al., 1999; Kojima et al., 2002; Lin et al., 2007; Schwartz et al., 2009	In many species promotes flowering.
4, 6, 7, 8, 9	13	Manes.13G001800	376245-380186	AP2-like ethylene responsive transcription factor	Cheng et al., 2023; Sgamma et al., 2014; Yant et al., 2010	In Chrysanthemum and *Arabidopsis*, represses flowering by direct repression of FT expression and involved in determining the identity of flower organs.
4, 6, 7, 8, 9	13	Manes.13G008300	815757-817107	TEOSINTE BRANCHED 1, cycloidea, PCF (TCP)-domain family protein 20	Colleoni et al., 2024; Dixon et al., 2018; Niwa et al., 2013	Key regulator of inflorescence architecture and flowering transitions. In *Arabidopsis*, TB1 plays a role in delaying floral transition by interfering with florigen (FT) function in the apical meristem. In wheat, TB1 interacts with FT and coordinates the formation of axillary spikelets during the vegetative-to-floral transition, affecting the timing and structure of flowering.
6, 7, 8, 9	13	Manes.13G009100	882446-883769	WRKY DNA-binding protein 75	(Song et al., 2024; Zhang et al., 2018b)	In *Arabidopsis* and *E. breviscapus*, it promotes flowering by activating FT expression in multiple pathways.
4, 6, 7, 8, 9	13	Manes.13G005200 Manes.13G022000 Manes.13G000200 Manes.13G026500	61249-61556 684757-686158 2122840-2124390 2462706-2465694	Pentatricopeptide repeat (PPR-like) superfamily protein	Bisht et al., 2025; Durand et al., 2021; Gaborieau et al., 2016	In several species is involved in restorers of cytoplasmic male sterility.
4, 6, 8	13	Manes.13G007100	768292-770229	exocyst subunit exo70 family protein	Marković et al., 2020	In *Arabidopsis*, it’s a component of the exocyst complex pollen that is required for efficient plant sexual reproduction.
4, 6, 8	13	Manes.13G007300	772559-773824	Pollen Ole e 1 allergen and extensin family protein	Alché et al., 2004	In *O. europaea* contributes to fertility by enabling key processes in pollen germination and fertilization.
4, 6, 8	13	Manes.13G007500	777594-778325	SAUR-like auxin-responsive protein family	Ren and Gray, 2015	In several species are key effector outputs of hormonal and environmental signals that regulate plant growth and development.
6, 7, 8, 9	13	Manes.13G008000	805359-808541	cyclophilin 20-2	Zhang et al., 2013	In *Arabidopsis*, modulates the conformation of BRASSINAZOLE-RESISTANT1, which binds the promoter of FLOWERING LOCUS D to regulate flowering.
7, 8, 9	13	Manes.13G011900	1124748-1126420	Protein TERMINAL FLOWER 1	Ahn et al., 2006; Goretti et al., 2020; Hanano and Goto, 2011; Nadal Bigas et al., 2025	In many species, it is a floral repressor, opposing the function of FLOWERING LOCUS T (FT).
7, 8, 9	13	Manes.13G014200	1378693-1382607	Protein phosphatase 2A regulatory B subunit	Heidari et al., 2013	In *Arabidopsis*, it is both a positive and negative regulator of flowering time, depending on the type of regulatory subunit involved.
7, 8, 9	13	Manes.13G018800, Manes.13G019000	1723356-1849503	beta-ketoacyl reductase 1	Beaudoin et al., 2009; Smirnova et al., 2013	In *Arabidopsis*, rice and tomato is critical for male fertility through its key function in fatty acid elongation necessary for pollen and anther development.
7, 8, 9	13	Manes.13G019500	1876657-1879104	Pectate lyase family protein	Zheng et al., 2018	In rice and *B. campestris* is associated with abnormal pollen wall development and partial male sterility, implicating its role in pollen formation and fertility.
7, 8, 9	13	Manes.13G020900	1990796-1997913	embryo defective 1745	Lee et al., 2021	In *Arabidopsis*, it is required for cell division and gamete viability.
8, 9	13	Manes.13G021100	2015956-2020637	Myosin heavy chain related	Jiang et al., 2007	In rice it controls pollen development by photoperiod-sensitive protein localizations.

## 4 Discussion

### 4.1 Phenotyping for flowering

The development of precise phenotyping methods is foundational to genetic studies. This work utilized a categorical 0–2 scoring scale for the absence, onset and presence of flowering, providing a simple yet effective tool to quantify the vegetative-to-reproductive transition in the AM1588 F_2_ population. Flowering scales have been utilized across diverse plant species to quantify this transition. For instance, in model species like Arabidopsis, detailed, multi-stage scales are used to precisely link visible flowering stages to the molecular expression profiles of key floral regulators (FT, FLC, SOC1) (Boyes et al., 2001). In barley developmental flowering stages determined are used in accordance with the Zadoks’ scale (Z55) (Parrado et al., 2024; Zadocks et al., 1974), while soybean defines flowering by the R1–R2 stages (beginning to full bloom) (Fehr et al., 1977; Plumblee and Harrelson, 2022). In the context of cassava, a presence and absence score of 1 and 0, respectively, has been used for flowering phenotyping (Fukuda et al., 2010). Our study employed a categorical 0–2 scoring scale to enhance resolution of the flowering phenotype for quantitative trait locus (QTL) mapping.

The time-series phenotyping provided crucial insights into the trait’s dynamics. The high correlation of 0.95 observed between later stages (8 MAP and 9 MAP) indicates that the plant’s final flowering status is largely established by eight months. Conversely, the lower correlation (0.54) between the earliest (4 MAP) and latest time point suggests that the genetic or environmental control governing early flowering initiation may be partially distinct from the factors regulating the established, later flowering phenotype, or more sensitive to environmental influence. In our analyses, there was perfect correlation between the presence of male and female flowers. The F_1_ parent of the mapping population was fertile, with both male and female flowers, despite having a male sterile donor (Bohorquez-Chaux et al., 2025). This may suggest that the male sterility trait is likely controlled by a recessive gene but remains to be investigated.

### 4.2 QTL identification and candidate genes

Efforts to identify genomic regions associated with flowering in cassava have so far focused primarily on the measurement of flowering-related traits, such as branching type and level, number of nodes, height to first branch, and the numbers of pistillate and staminate flowers (Baguma et al., 2024; Boonchanawiwat et al., 2011; Ewa et al., 2021; Zhang et al., 2018a). However, unlike previous efforts that focused on secondary traits or proxies (such as branching architecture), this study represents the first reported QTL mapping analysis directly based on a categorical flowering time scale (the 0–2 scoring system).

This study successfully identified and validated QTL associated with flowering in cassava, providing critical genomic resources for accelerating breeding efforts in this staple crop. Our results confirm the quantitative and polygenic nature of flowering time in cassava, consistent with other crops, while highlighting a major-effect locus on chromosome 13 (*Meflwr13*) as the primary determinant of flowering variation within the AM1588 F_2_ population. The identification of *Meflwr13*, which consistently exhibited the highest LOD score and explained the largest proportion of phenotypic variance (up to 42.63%) across all five evaluation time points (4, 6, 7, 8, and 9 MAP) was key. The stability and magnitude of this QTL strongly suggest it harbors major genes controlling flowering. Besides *Meflwr13*, we also identified other QTL for flowering on chromosomes 1, 7 and 16. *Meflwr1* was consistent across four time points, indicating a role in general flowering initiation, albeit with a smaller phenotypic variation explained (up to 13.26%). The region harbors an APRR5 response regulator homolog (Manes.01G043200), which is part of the circadian clock. It is known to regulate flowering time by influencing CO/FT expression (Hayama et al., 2017; Sato et al., 2002). *Meflwr7* was mapped at 4, 6 and 7 MAP with R^2^ values up to 13.18%, suggesting it modulates early-to-mid stage reproductive development before its influence diminishes in later stages. *Meflwr16* was only mapped at one time point (8MAP) suggesting it is likely transient or more stage-specific, and may function in a more environment-specific manner.

*Meflwr13* QTL encompasses two genes central to the global floral network: Manes.13G000800, a homolog of FLOWERING LOCUS T (FT) and Manes.13G011900, a homolog of TERMINAL FLOWER 1 (TFL1) (**Figure 4**). Both FT and TFL1 belong to the phosphatidylethanolamine-binding protein (PEBP) family and are highly homologous (Ahn et al., 2006; Colleoni et al., 2024; Nadal Bigas et al., 2025). Despite this structural similarity, they perform opposite functions in plants (Azevedo et al., 2025; Jin et al., 2021; Kaneko-Suzuki et al., 2018; Liu et al., 2021; Matsoukas et al., 2012). FT acts as a florigen, promoting flowering, while TFL1 is a floral repressor by antagonizing the activity of FT. This antagonistic relationship is critical in regulating the transition from vegetative growth to reproductive phase. The proximity of these genes in the QTL—and the functional relationship between the FT and TFL1 orthologs—indicates a high likelihood of functional interactions between these genes in the region to control the flowering phenotype. This co-localization supports previous functional studies in cassava that demonstrated the central role of FT homologs in controlling flowering and branching (Adeyemo et al., 2011, 2017, 2019).

This regulatory complexity is further supported by the inclusion of two key transcription factors that control the core FT/TFL1 switch: Manes.13G001800, an AP2-like ethylene-responsive transcription factor (AP2/ERF TF), and Manes.13G008300, which encodes a TEOSINTE BRANCHED 1 (TB1) gene. The AP2/ERF TFs are known to act as transcriptional repressors of flowering, often directly inhibiting FT gene transcription in the leaves to reduce the florigen signal (Sgamma et al., 2014; Yant et al., 2010). Similarly, TB1 is a founding member of the TCP (TEOSINTE BRANCHED 1/cycloidea/proliferating cell factors) family of transcription factors that are critical regulators of plant architecture. TB1 and related TCP proteins have been described to interact directly with FT in other crops including apples (Mimida et al., 2011), rye (Zhan et al., 2023), and *Brassica juncea* (Feng et al., 2022). Their primary mechanism is thought to involve disrupting the florigen activation complex, thereby delaying flowering.

Additionally, the region includes a WRKY75 transcription factor (Manes.13G009100), identified near the most significant SNP from QTL mapping. Various WRKY TFs have been identified to play a crucial role in modulating flowering pathways to regulate flowering time (Song et al., 2024). Specifically, WRKY75 orthologs have been repeatedly implicated in promoting flowering across different species. In *Brassica juncea*, *Bju*WRKY75 promotes flowering by activating FT expression (Feng et al., 2022). In the gibberellin pathway, AtWRKY75 binds to FT to promote its expression, resulting in early flowering (Zhang et al., 2018b). Furthermore, overexpression of *Cp*WRKY75 from *Chimonanthus praecox* is involved in multiple pathways to promote flowering time in transgenic Arabidopsis (Huang et al., 2022). Studies established that both *Md*FT1 and *Md*TFL1 interact competitively with *Md*WRKY6 protein to facilitate and inhibit, respectively, activation of an apple *LEAFY*-like gene, ultimately regulating apple flower bud formation (Zuo et al., 2024). The presence and proximity of these antagonistic and promoting factors in *Meflwr13* indicates a highly complex and integrated molecular switch driving the primary flowering phenotype.

### 4.3 Implications for fertility and pleiotropy

The genetic background of the mapping population, derived from a male-sterile grandmother (ECU72), adds a layer of complexity. Although the F_1_ parent was fertile, the subsequent perfect correlation between male and female flower presence in the F_2_ is noteworthy. The identification of several genes related to sterility, pollen development, and fertility restoration within the *Meflwr13* interval (**Table 3**)—such as PPR repeats, beta-ketoacyl reductase 1, and pectate lyase (Beaudoin et al., 2009; Bisht et al., 2025; Durand et al., 2021; Gaborieau et al., 2016; Liu et al., 2017; Smirnova et al., 2013; Zheng et al., 2018) —suggests a possible pleiotropic role for this locus. However, it remains to be determined whether this arises from true biological pleiotropy or tight linkage drag between flowering time regulators (e.g., FT/TFL1) and distinct fertility genes. This makes the region a strong candidate for future investigation into the genetic basis of male sterility and fertility restoration in cassava, which could further refine breeding strategies. The region could be further dissected through high-resolution fine-mapping by increasing marker density within the interval and screening a larger F_2_ or F_3_ population to identify recombinants that potentially uncouple these traits.

### 4.4 Marker identification, validation and application

Marker selection focused on SNPs within the peak regions to confirm their association with the trait, with the *Meflwr13* SNPs exhibiting the strongest statistical association (**Figure 2**). This high association was successfully confirmed by validation in the independent progenitor population (**Figure 3**), which is a crucial step toward practical application. The analysis revealed that three of the six key SNPs—Chr13_889929, Chr13_634483, and Chr13_658450—are the most robust candidates. They exhibited a dominant segregation pattern for the favorable (flowering) allele and showed the most acceptable False Negative Rate (FNR) values (**Table 2**). While these markers exhibited higher FPR compared to the excluded markers, they provide the most effective compromise for breeding, prioritizing the retention of favorable flowering alleles. In contrast, the alternative markers displayed FNR values exceeding 60%, which would result in the inadvertent discarding of the majority of favorable genotypes. Dominant markers are highly prized in breeding because they simplify the selection process. While the validation in the diverse progenitor population demonstrates the utility of these markers, their broader applicability across distinct genetic backgrounds and geographic locations warrants further investigation to ensure stability. Flowering is heavily influenced by environmental cues such as photoperiod, temperature, and altitude. It will be essential to determine if the *Meflwr13* QTL effect remains stable or if significant genotype-by-environment interactions influence the efficacy of marker-assisted selection.

Current flower-induction technologies including photoperiod extension, branch pruning, and the application of growth regulators are highly labor-intensive and costly to implement. For instance, expenses may range from approximately USD 1,000–3,000 for a dozen progenitors in a national breeding program. Furthermore, the effectiveness of these manual methods has only been evaluated in a limited number of genotypes, underscoring the urgent need for robust, large-scale genetic tools (Barinas et al., 2023).

The successful validation of these dominant markers within the major *Meflwr13* locus has immediate and significant translational implications. Developing molecular markers that enable early selection—at the nursery stage— is the ideal solution. Breeders can use these markers in marker-assisted selection (MAS) to rapidly and cost-effectively screen seedlings before they are transplanted to the field. Early flowering genotypes, confirmed by the presence of the favorable dominant *Meflwr13* allele, can be immediately allocated to standard crossing lots, saving time and resources by eliminating the need for expensive flower-induction treatments. These resources can then be strategically redirected toward those genotypes expected to flower late, maximizing the efficiency of the entire breeding pipeline.

## 5 Conclusion

This study has localized a robust, major-effect QTL for flowering in cassava to chromosome 13, pointing directly to the FT/TFL1 gene region as a primary genetic control switch. The validated SNP markers provide tools for MAS to improve flowering reliability, a prerequisite for the efficient genetic improvement of cassava.

## Data Availability

The datasets presented in this study can be found in online repositories. The names of the repository/repositories and accession number(s) can be found in the article/**Supplementary Material**.
