# Supplementary figures and images for: A major FT/TFL1 regulatory locus (Meflwr13) controls flowering time in cassava and provides validated markers for accelerated breeding
Source: Front Plant Sci. 2026 Feb 17;17:1741780. doi: 10.3389/fpls.2026.1741780 (PMC12953470; doi:10.3389/fpls.2026.1741780)

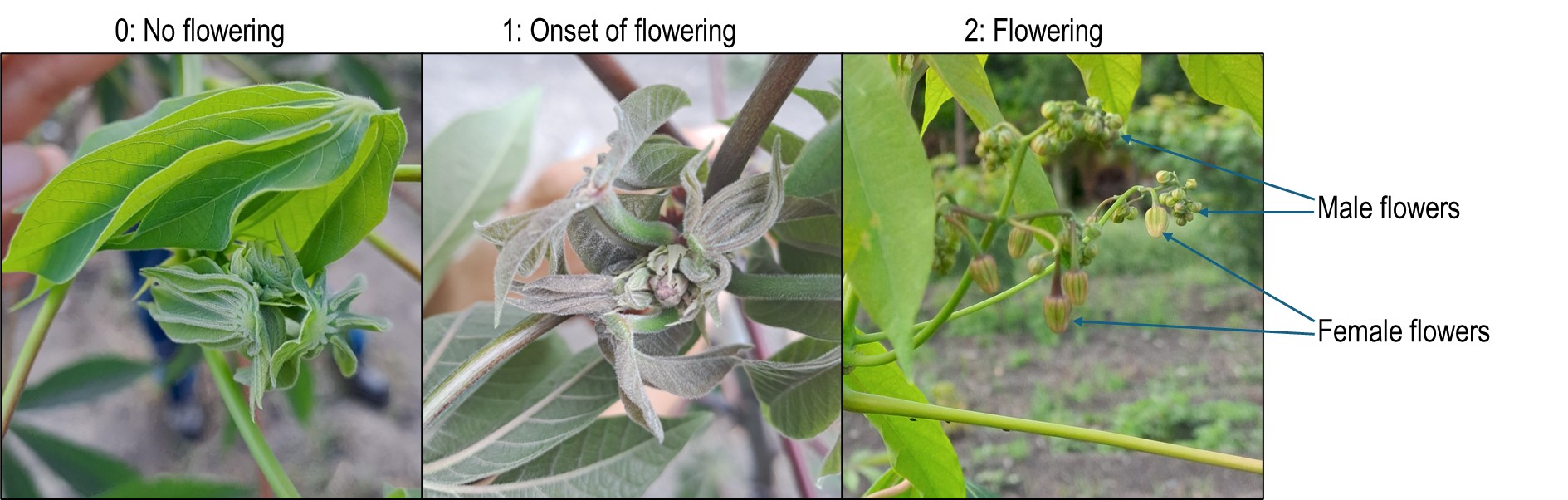

Supplement: Supplementary file 1 [file Image1.jpeg]

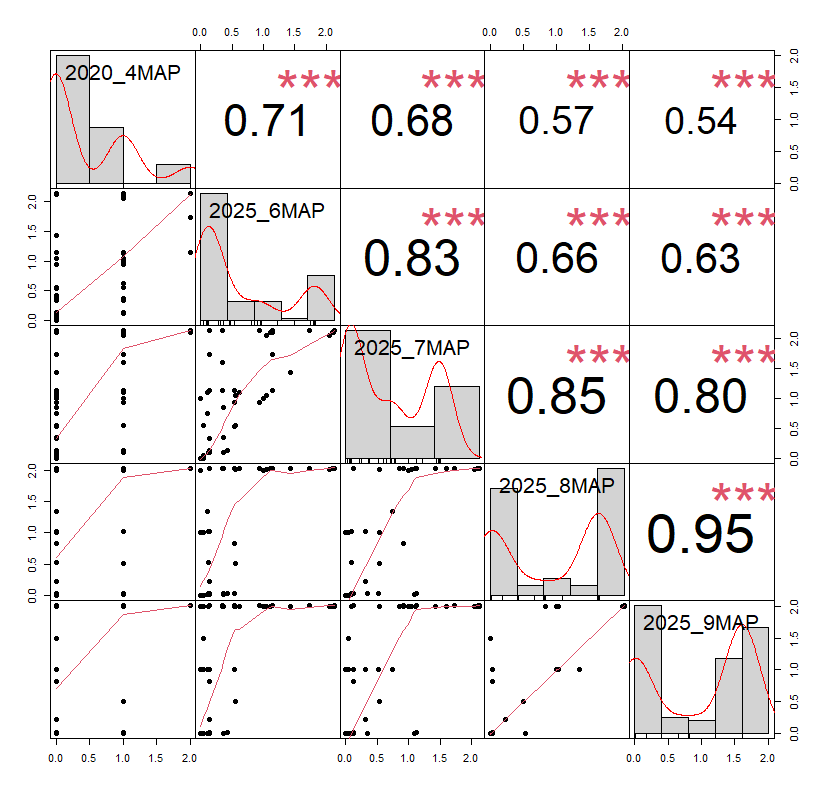

Supplement: Supplementary file 2 [file Image2.tiff]
